# Double-edged sword of interdisciplinary knowledge flow from hard sciences to humanities and social sciences: Evidence from China

**DOI:** 10.1371/journal.pone.0184977

**Published:** 2017-09-21

**Authors:** Meijun Liu, Dongbo Shi, Jiang Li

**Affiliations:** 1 Division of Information and Technology Studies, the University of Hong Kong, Hong Hong, China; 2 School of International and Public Affairs, Shanghai Jiao Tong University, Shanghai, China; 3 School of Economics and Trade, Hunan University, Changsha, China; 4 Department of Information Resource Management, Zhejiang University, Hangzhou, China; Institut Català de Paleoecologia Humana i Evolució Social (IPHES), SPAIN

## Abstract

Humanities and Social Sciences (HSS) increasingly absorb knowledge from Hard Sciences, i.e., Science, Technology, Agriculture and Medicine (STAM), as testified by a growing number of citations. However, whether citing more Hard Sciences brings more citations to HSS remains to be investigated. Based on China’s HSS articles indexed by the Web of Science during 1998–2014, this paper estimated two-way fixed effects negative binomial models, with journal effects and year effects. Findings include: (1) An inverse U-shaped curve was observed between the percentage of STAM references to the HSS articles and the number of citations they received; (2) STAM contributed increasing knowledge to China’s HSS, while Science and Technology knowledge contributed more citations to HSS articles. It is recommended that research policy should be adjusted to encourage HSS researchers to adequately integrate STAM knowledge when conducting interdisciplinary research, as over-cited STAM knowledge may jeopardize the readability of HSS articles.

## Introduction

Interdisciplinary research has been long regarded as a catalyst for breakthroughs and innovations, as well as an effective tool to address increasingly complex socio/economic problems and foster competitiveness [1]. Since the 1960s, interdisciplinary issue has become a major topic in discourse on knowledge creation and research funding [2]. Many science policies encouraged interdisciplinary research with high expectation by creating multidisciplinary centers and increasing the amount of funding targeted at boosting interdisciplinary collaboration [3]. Driven by policies and funding preferences, research has become ever more interdisciplinary. Since the mid-1980s, research papers have increasingly cited work outside their own disciplines [4].

It is beneficial for the understanding of interdisciplinary knowledge flow to explore the impact of integrating knowledge from Hard Sciences (HS), including Science, Technology, Agriculture and Medicine (STAM), into Humanities and Social Sciences (HSS). It is widely acknowledged that there exists a huge knowledge gap between HSS and HS due to their differences in subject matter and fundamental relationship, unified paradigms, multilateral theories and so forth [5]. Furthermore, interdisciplinary knowledge flow from HS to HSS influences science policy towards research funding. In the past few years, data availability has advanced the quantitative aspects of HSS research. Preferential science policies encouraged interdisciplinary research which integrated knowledge from both HSS and HS.

Does citing more HS bring more citations to HSS? In order to address this research question, we explore the relationship between the ratio of HS references in HSS articles and the number of citations they received, by examining China’s HSS articles indexed by the Web of Science during 1998–2014 and using bibliometric techniques and regression analysis. The paper is organized as follows: next section reviews related literature on knowledge flow, interdisciplinarity and citations, and the interaction between HS and HSS; the following section explains the data and methodology in detail; the fourth section presents the results; and in the final section, we provide discussion and conclude our findings.

## Literature review

### Knowledge flow

Citations from HSS to HS indicate the diffusion of interdisciplinary knowledge. Knowledge diffusion, or spread of scientific ideas, is a virtual movement through cognitive space over/through different units. It is a determining factor of the innovativeness of the ideas spread [6]. There are multiple forms of knowledge diffusion, such as citations, collaboration among individuals or research groups, etc. [7]. Also, the units of knowledge diffusion include journals, subject categories, institutes, countries, etc. [8–11]. As an important carrier of authors’ scientific ideas/knowledge, publications borrow knowledge from previous ones and diffuse to the future ones. This process forms a chain of knowledge flow, whereby knowledge flows from cited references to citing publications via citations. In the process of knowledge flow, some disciplines are “donors”, others “receptors” [12]. For example, Management was deemed as a crucial donor for Psychology and its main information is acquired from Economics, Psychology and Sociology [13]. Sociology and Education imported many ideas from other disciplines, while Psychology, Linguistics, Philosophy and History exported knowledge to other disciplines [14].

### Interdisciplinarity and citations

Interdisciplinary research brings creativity, progress, innovation and even intellectual “breakthroughs” [15]. Efforts were devoted to measuring interdisciplinarity of articles, authors or journals, mainly through citations, collaboration or networks. Pratt index was introduced to measure the concentration and scattering of documents [16]. Citation Outside Category (COC) was proposed as a measure of percentage of citations outside a specific discipline [17]. However, this indicator is controversial since it fails to offer a complete and precise account of citation data [18]. Brillouin’s Index, compared with these two approaches, has been given more favor, since it integrated richness and relative abundance into a single index value [18]. In addition, network coherence was used to capture interdisciplinarity and reflect the novelty of its knowledge integration [19]. Recently, a new method was proposed to measure interdisciplinarity by means of adding a component that captures connectivity between subject categories [20].

The relationship between interdisciplinarity and the number of citations has attracted considerable attention. The concept of diversity, including variety, balance and disparity [21], is used to measure interdisciplinarity. The diversity of interdisciplinary knowledge of an article is often mirrored by the diversity of its references [22, 23]. An inverted U-shape relationship was found between interdisciplinary degree and citation, as well as a positive effect of variety on citation while balance and disparity were proved to have a negative effect [24]. However, the authors illustrated limitations of the measurement in their research, inaccuracies in the Web of Science (WoS) categories, potential differences in citing behavior between the studied disciplines and others, and selection of the 5-year citation window and controls in the model. By contrast, some researchers offered empirical evidence that variety and disparity have positive effects on long-term citations and negative effects on short-term citations [25]. Interdisciplinary research in life science, health science, and physical science receive fewer citations than monodisciplinary counterparts, although this finding is not applicable to social sciences [3].

In spite of these positive influences, there are some “costs” to interdisciplinarity, e.g. requiring strenuous efforts, and reducing the chances of success [26]. From the perspective of interdisciplinary collaboration, the cost of interdisciplinary research can be categorized into coordination costs and institutional costs. The difficulties of knowledge integration can result in coordination costs due to the lack of a common language, shared meanings and norms, negotiations to harmonize differences in management and culture, and administrative load and time [27, 28]. Institutional costs arise from institutionalization of science in terms of disciplines [24]. Previous studies demonstrated that there are four main costs on interdisciplinary collaboration, i.e. poor structure for scientific interdisciplinary researchers, low esteem by colleagues, difficulty to publish in prestigious journals and discrimination by reviewers [29].

With the appearance and development of interdisciplinary research fields, the interaction between HSS and HS was gradually emphasized [30–33]. Many studies attempted to explore how HS research or programs learned from HSS, such as analysis methods, management tools and theories, to make it run smooth or grow more socially-robust [34–36]. However, some impediments hinder the participation of HSS with HS. Major barriers include weakness and perceived illegitimacy of HSS, punishments for interdisciplinary involvement, the scarcity of a disciplinary support structure and issues of power and control [37]. Moreover, from the perspective of attitudes of both sides, the lack of respect between HSS and HS also hampers the integration [33].

Based on previous literature, as a kind of interdisciplinary knowledge flow with both benefits and costs, citing HS knowledge in HSS articles may also have both positive and negative effects on citations of HSS articles, which means that an optimal degree of HS references for high citations may exist. Therefore, we put forward the following hypothesis:

In the field of HSS, citing HS publications has an inverted U-shaped effect on the number of citations that HSS articles receive.

## Material and methodology

### Data and measurement

In China, HS was preferred in terms of both public policy and R&D expenditure in the 1980s and the 1990s [38]. By the end of the 1990s, China’s HSS started to be innovative and line up with international academic norms [39]. Compared with the fast development of China’s Science Citation Index (SCI) publications [40], the number of English HSS papers appeared lackluster although the annual number of publications indexed by the Social Science Citation Index (SSCI) or the Arts and Humanities Citation Index (A&HCI) increased exponentially in the past two decades [41, 42]. The vast majority of China’s output in HSS was still published in Chinese, and hence lacks international visibility.

The raw data consists of 69,746 papers with 2,355,376 references, published by at least one Chinese institution during the period 1998–2014 and indexed by SSCI or A&HCI. The data of this study was extracted from WoS, by the following queries,

*ADDRESS*: *(People R China)*
*Timespan*: *1998–2014*. *Indexes*: *SSCI*, *A&HCI*

The **proportion of HS references** in an article is defined as follows:

$${STAMP}_{i}=\frac{{HSR}_{i}}{{ALLR}_{i}},$$ (1)

where *HSR*_*i*_ denotes the number of references of article *i* classified into Science, Technology, Agriculture or Medicine, and covered by the WoS, and *ALLR*_*i*_ represents the total number of references of article *i*.

Based on the WoS subject category, we attempt to assign a unique discipline to every publication. The WoS, which indexed 11,813 journals in 2014, sorted all journals into 232 sub-disciplines. In WoS, it is possible for one journal, except multidisciplinary ones, to be categorized into more than one subject, which hinders interdisciplinary citation analysis. In this case, it is reasonable to classify the journal into the discipline where it received the largest number of citations. Then, each paper in our dataset and its references have a unique disciplinary affiliation based on the subject of journals where articles were published.

It should be noted that most categories in HS are far larger than the ones in HSS in terms of the number of journals and publications. If a journal belongs to a small discipline of humanities, it may be hardly possible for this journal to receive the largest number of citations from its home discipline. Even though there is a limitation that the size of disciplines is not considered in terms of our classification efforts, given that we focus on HSS or HS category of articles and their references, the unique category of publications is reliable. This is because according to the WoS category, only 7% of the total number of journals indexed by WoS were assigned to both HS and HSS subjects. For example, Journal of Informetrics was categorized into both Computer Science and Information Science & Library Science. Accordingly, using citation-based methods to address multi-category problems may not be problematic when we pay more attention to the HSS or HS category of articles rather than more specific categories of them (e.g. sub-disciplines in HSS or those in HS).

The next step is to categorize the 232 subjects into the 12 MOE (Ministry of Education of the People’s Republic of China) disciplines by utilizing the Library of Congress Classification Outline [43] which bridges the WoS categories and the MOE disciplines. The disciplines we mention in this research are the twelve disciplines from the Ministry of Education of People’s Republic of China [44], i.e., Language & Literature, History, Philosophy, Arts, Economics, Education, Law, Management, Science, Technology, Agriculture and Medicine, which contain 110 sub-disciplines in total. In this study, Language & Literature, History, Philosophy and Arts are referred to as ‘Humanities’, Economics, Law, Management and Education as ‘Social Sciences’, and Science, Technology, Agriculture and Medicine as ‘Hard Sciences’. As a result, our final dataset contained 31,335 HSS articles and 1,315,497 references. Among the 31,335 HSS papers, 4.96% are on Humanities and 47.60% are internationally coauthored. Among the 1,315,497 references, 54.6% are indexed by the Web of Science, with the rest not considered in this research.

### Variable operationalization and models

The percentage of HS references to all references indexed in WoS in HSS articles is defined as the independent variable (*STAMP*). Referencing previous studies [24, 45–48], we choose the following variables as controls: the ratio of references which are self-citations at discipline level to references indexed in WoS (*Selfciting*), the number of authors of each article (*Author*), the number of keywords of each article (*Keyword*), the number of pages of each article (*Page*), the number of references of each article (*Reference*). Besides, whether an article is internationally coauthored is a dummy variable (*International*): 1 if yes, 0 else. When we did robustness check, the percentage of HS references indexed in WoS was substituted for the number of HS references (*STAMN*).

It is necessary to compare citations within the same citation window. The dependent variables, the short-term (*Win3all*) and long-term (*Win10all*) scientific impact, are defined as the citation counts of each article in a three-year and a ten-year citation window, respectively [24, 25, 45, 49] based on the consideration that the number of citations an article received never decreases, and usually increases with time. The subsample in which HSS articles are published between 1998 and 2004 of dataset allows us to obtain 10-year citation counts of HSS articles, including 3,920 articles. Furthermore, we also considered who cited HSS articles so that overall citation counts are divided into citations from HSS *(Win3hss*, *Win10hss*) and those from HS (*Win3hs*, *Win10hs*) in both a short and long term.

Two-way fixed effects negative binomial models are used to test the relationship between citing HS knowledge and the citation HSS received. Poisson regression or negative binominal regression is applicable to this study, since the dependent variables are count variables [50–52]. Poisson regression works on condition that the variance of the variable equals to the average, due to the mean-variance equality [53]. As shown in Table 1 and Table 2, the variance of the dependent variable exceeds the mean so that to address the research question a negative binomial model should be adopted. In order to eliminate the quality between different articles, we drew on previous studies [25, 54] and considered journals fixed-effects. Whether journal fixed effects or random effects should be taken into account depends on whether the intercept of unobserved individual heterogeneity is correlated to explanatory variables. Previous studies revealed that the impact factor of the journal in which an article was published was correlated to the international co-authorship of the article [55, 56]. Furthermore, self-citations were more prevalent in journals with lower impact factor [57]. Hence, the intercept of unobserved heterogeneity of articles may be related to some explanatory variables in this study, i.e. *International* and *Self-citing*. Therefore, we conducted a Hausman specification test [58]. Results showed that the journal fixed effects model was more efficient and more appropriate than the random effect model, in estimating the effect of HS references on the short-term or the long-term citations to HSS papers. In addition, year dummies were added into models to overcome time effects. Then, a two-way fixed-effects model including journal and year dummies was estimated.

**Table 1:** Descriptive statistics and pearson correlation coefficient of variables in the short-term citation window (three-year)(N = 31,335).

Variable	Mean	Std.Dev.	Min	Max	1	2	3	4	5	6	7	8	9	10
**1.Win3hss**	1.376	2.795	0	139										
**2.Win3hs**	0.637	1.9	0	65	0.283									
**3.Win3all**	2.013	3.799	0	156	0.877	0.709								
**4.STAMP**	0.188	0.262	0	1	-0.075	0.238	0.064							
**5.STAMN**	4.276	7.791	0	177	0.001	0.309	0.155	0.703						
**6.Selfciting**	0.624	0.305	0	1	0.069	-0.133	-0.016	-0.662	-0.468					
**7.Internationl**	0.524	0.499	0	1	0.11	0.036	0.099	-0.016	0.04	0.025				
**8.Author**	3.177	3.851	1	396	0.055	0.094	0.087	0.186	0.191	-0.119	0.172			
**9.Keyword**	3.752	3.066	0	45	0.009	0.059	0.036	0.076	0.121	-0.074	0.029	0.138		
**10.Page**	15.51	8.197	0	335	0.06	-0.074	0.007	-0.229	-0.108	0.118	0.07	-0.083	-0.036	
**11.Reference**	41.98	26.05	0	409	0.183	0.048	0.159	-0.078	0.245	0.023	0.119	0.039	0.122	0.406

**Table 2:** Descriptive statistics and pearson correlation coefficient of variables in the long-term citation window (ten-year) (N = 3,920).

Variable	Mean	Std.Dev.	Min	Max	1	2	3	4	5	6	7	8	9	10
**1.Win10all**	12.88	23.79	0	342										
**2.Win10hss**	10.15	18.1	0	285	0.935									
**3.Win10hs**	3.976	8.233	0	111	0.751	0.502								
**4.STAMP**	0.158	0.256	0	1	-0.011	-0.131	0.213							
**5.STAMN**	2.67	5.894	0	132	0.072	-0.047	0.276	0.646						
**6.Selfciting**	0.666	0.314	0	1	-0.021	0.047	-0.131	-0.647	-0.417					
**7.Internationl**	0.475	0.499	0	1	0.197	0.198	0.133	-0.015	0.028	0.021				
**8.Author**	2.558	3.51	1	131	0.117	0.104	0.095	0.081	0.089	-0.052	0.204			
**9.Keyword**	2.109	2.565	0	15	0.022	-0.019	0.096	0.104	0.096	-0.061	0.013	0.027		
**10.Page**	16.44	9.746	0	335	0.076	0.119	-0.011	-0.176	-0.033	0.070	0.069	-0.016	0.005	
**11.Reference**	37.05	25.22	1	409	0.199	0.220	0.104	-0.050	0.294	-0.011	0.052	0.062	-0.018	0.465

To test the hypothesis, we constructed an equation which incorporated all variables described above. Besides linear relationship between the ratio of HS references and the number of citations HSS articles received, we are also concerned with the potential curvilinear relationship between them, so the squared term of the independent variable was added, considering both positive and negative effects of interdisciplinary knowledge flow in previous literature. Besides, we incorporated not only journal fixed effects but also year fixed effects to estimate within-journal effects and within-year effects as shown in Eq 2.

$$Citation=f(\alpha +\beta_{1}STAMP+\beta_{2}{STAMP}^{2}+\beta_{3}Selfciting+\beta_{4}International+\beta_{5}Author+\beta_{6}Keyword+\beta_{7}Page+\beta_{8}Reference+\beta_{9}\;Journal+\beta_{10}Year+\varepsilon )$$ (2)

## Results

### The increasing contribution of HS to HSS

**Fig 1:**
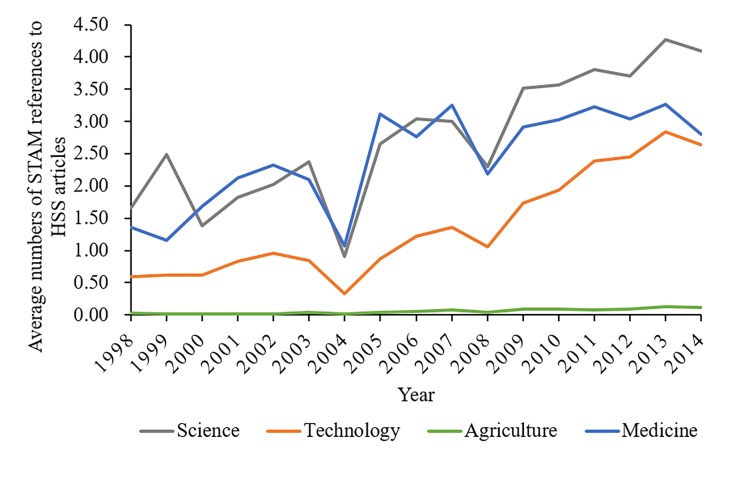
The longitudinal trend of the average numbers of STAM references to HSS articles.

It is clear that STAM contributed more and more knowledge to HSS through the years 1998–2014, as shown in Fig 1. At the beginning of the period, on average each HSS article cited only 1.66 Science, 0.59 Technology, 0.03 Agriculture and 1.36 Medicine publications, respectively. However, the numbers increased to 4.09, 2.63, 0.12 and 2.80 by 2014. Note that the numbers of STAM references would be significantly higher, if we counted in the STAM references which are not indexed by the Web of Science. It is observed that Science, Technology and Medicine were major knowledge bases for HSS over the whole period, while citing Agriculture seems least frequent.

**Fig 2:**
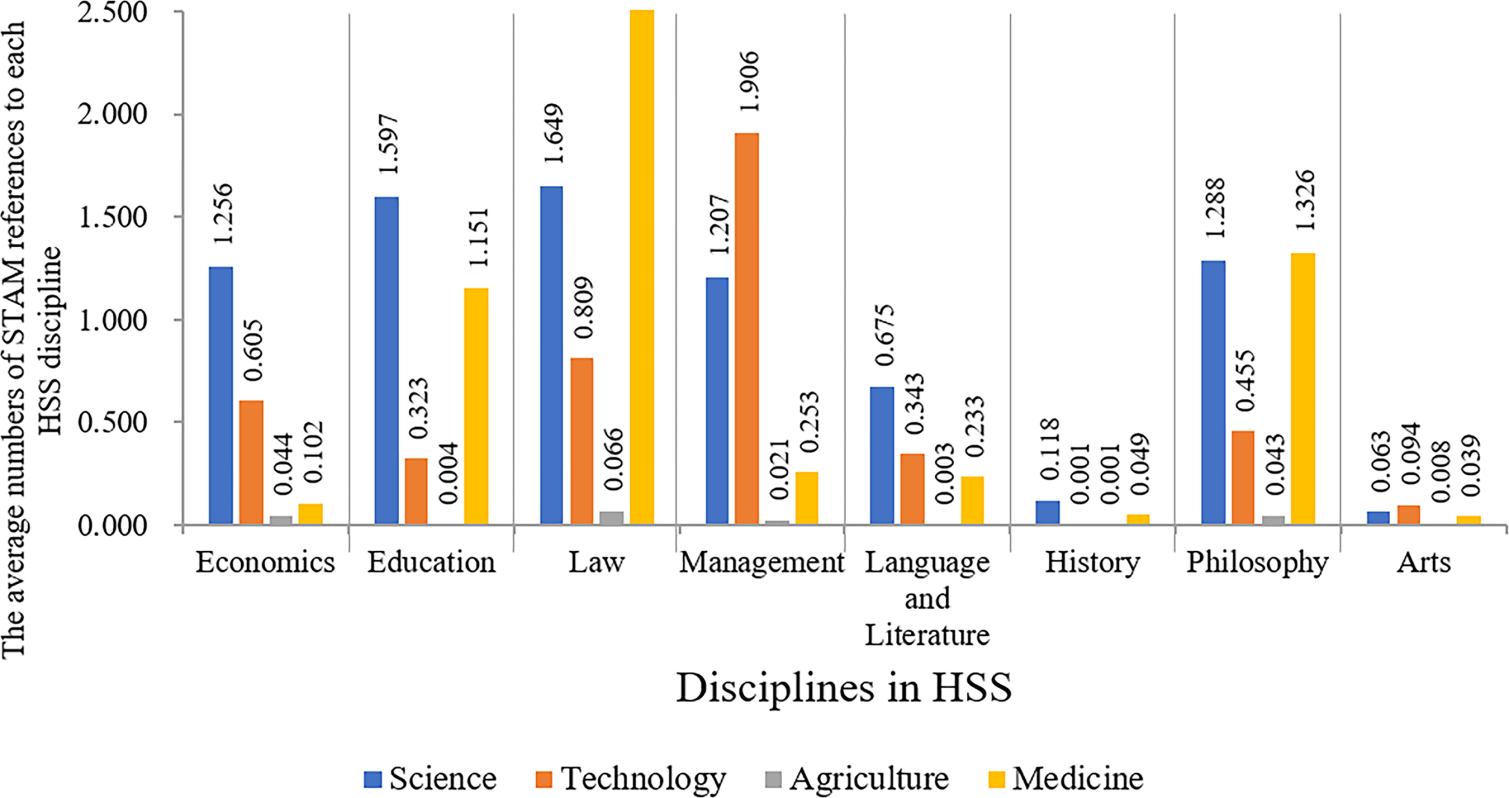
The average numbers of STAM references to each HSS discipline.

The knowledge contribution from STAM to HSS is heavily skewed at discipline level. Fig 2 presents the average numbers of STAM references to each HSS discipline, indicating that HS contributed more knowledge to Social Sciences than to Humanities. Through the years 1998–2014, on average each Social Sciences article cited 3.58 HS articles, far exceeding the number of Humanities, i.e., 1.34. Social Sciences preferred Science to other HS disciplines. On average, each Social Sciences article cited 1.43 Science papers, but only 1.00 Medicine, 0.91 Technology and 0.03 Agriculture papers, respectively. Management absorbed more Technology knowledge than did other Social Sciences. For example, on average each Management article cited 1.91 Technology papers, but for Law and Economics, the numbers are 0.81 and 0.60, respectively. It seems Law prefers Medicine knowledge, compared to other Social Sciences, i.e., each Law article cited 2.50 Medical papers on average. Humanities showed less interest in HS knowledge. On average each Humanities article cited merely 0.54 Science, 0.23 Technology, 0.02 Agriculture and 0.41 Medicine papers, respectively. Nevertheless, compared to other Humanities, Philosophy absorbed significantly more HS knowledge, especially from Science. For example, on average each Philosophy article cited 1.29 Science papers.

### An inverse U-shape curve between the percentage of HS references in the HSS articles and the number of citations they received

**Fig 3:**
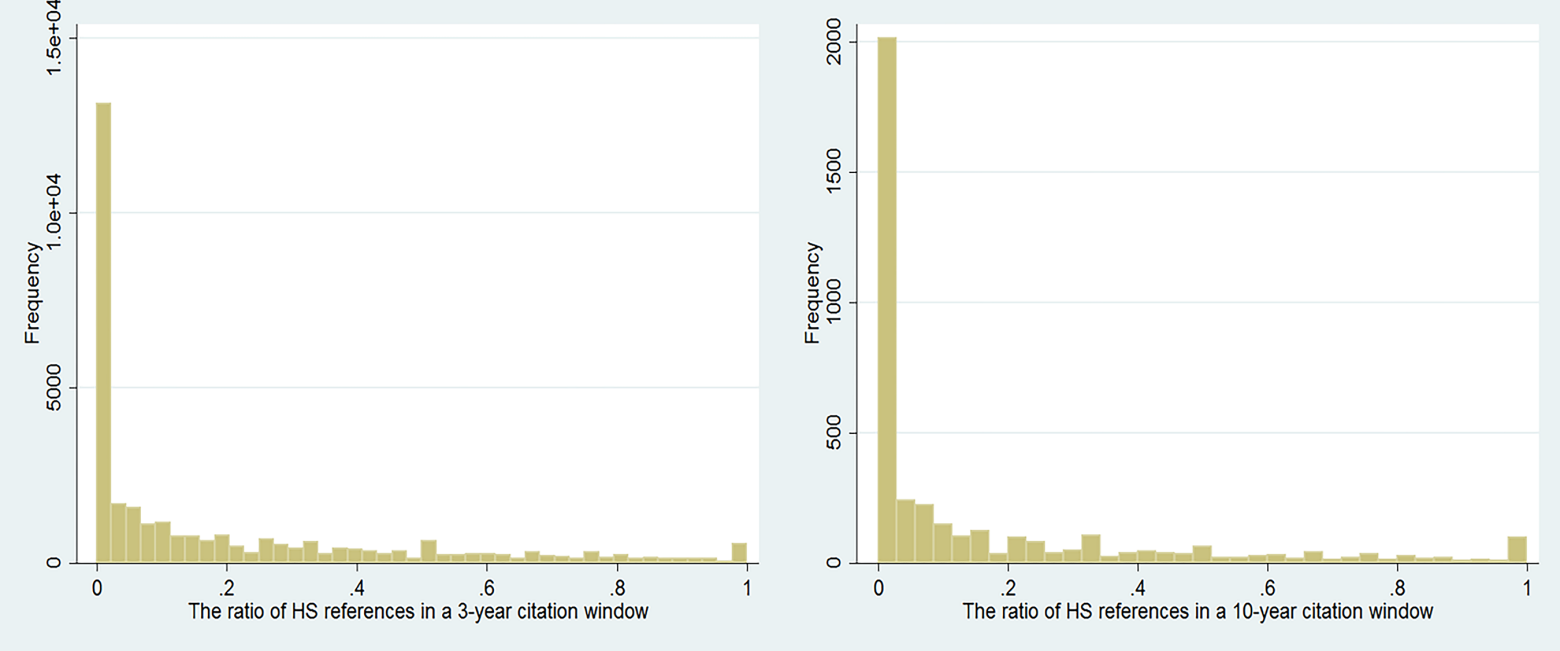
The distribution of the percentage of HS references in HSS article.

Table 1 and Table 2 show that the explanatory variables are not highly correlated among themselves in addition to the correlation (-0.662) between *Self-citing* and *STAMP* in Table 1. Then, we calculated the mean variance inflation factor score (1.61), which is far lower than critical point 10, signifying that multicollinearity does not trigger any problem in this study; therefore, the regression results are consistent and unaffected by the high correlations between these explanatory variables [59]. In addition, the distribution of the percentage of HS references in HSS articles is reported in Fig 3.

**Fig 4:**
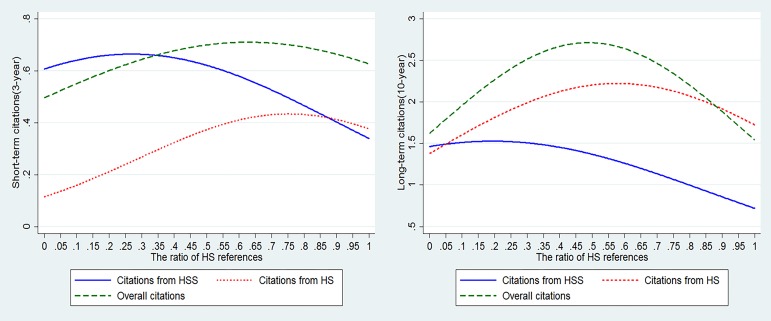
Relationship between the estimated values of citation impact and the ratios of HS references.

It is observed that an inverse U-shaped curve exists in the relationship between the percentage of HS references and the number of citations HSS articles received, regardless of whether the citation window is short-term or long-term. We first estimated fixed effects Negative Binomial models using the short-term citation counts (3-year) as the dependent variable and the ratio of HS references as the independent variable, as shown in Table 3. Overall citation counts can be decomposed into citation counts from HS and those from HSS. From column 1 to column 2, we did not differentiate the disciplinary attribute of citations, using overall citation counts as the dependent variable. Then, column 3 to column 4, and column 5 to column 6 where citation counts from HSS and those from HS as dependent variables respectively, reported the regression results. For each type of citation counts, we first introduced *STAMP* to test the possible effect of the proportion of HS references on HSS articles’ citations. Subsequently, the squared term *STAMP*2 was included. Column 1 to Column 2 shows that there is a significant inverted U-shaped relationship between *STAMP* and the overall number of citations HSS papers acquired as the coefficient for *STAMP* is positive (β = 1.144, p<0.01) and that for *STAMP*2 is negative (β = -0.909, p<0.01). This result indicates that citing more Hard Sciences brings more overall citations to HSS papers before the ratio of HS references reaches a turning point. Then, from column 3 to column 4, citation counts from HSS are regarded as dependent variables. It seems that *STAMP* has negative influence on the citation from HSS per se with a significantly negative coefficient (β = -0.265, p<0.01). But when *STAMP*2 was added in column 4, an inverted U-curve still exists. Furthermore, we also turned our attention to the impact of *STAMP* on the citation counts from HS and found an inverted U-shaped relationship again, which suggests that more Hard Sciences references may help increase citation counts from HS on condition that the percentage of HS references is less than the number at the turning point. In order to explore how the percentage of HS references functions in a long-term citation window (10-year), we ran regression and did the same regression analysis as reported in Table 4. It appears that inverted U-shape curves are also found between the proportion of HS references and overall citation counts, citation from HSS and those from HS. For better visual presentation, the estimated citation counts in short-term and long-term citation windows by the ratio of HS references are plotted in Fig 4. Consistent with Wang’s study [25], based on models including STAMP and STAMP2, these estimations hold *international* at 0, fixed effect being 0 and all other variables at their means. In the short run, it is found that after the ratio of HS references reaches 27%, the positive effect on citations from HSS may turn negative reversely. As to models based on citations from HS as dependent variables, the turning point is approximately 75% far higher than the one just mentioned. Furthermore, for HSS articles to obtain more overall citation counts, the percentile of HS references should be controlled within 63%. In addition, in the long-term citation window, the turning points in models based on citations from HSS, those from HS and overall citation counts decline to 19.35%, 57.80% and 48.86% separately.

**Table 3:** Fixed effects negative binomial models: The percentage of hard sciences references and short-term citation (three-year) (N = 31,335).

	(1)	(2)	(3)	(4)	(5)	(6)
	Win3all	Win3all	Win3hss	Win3hss	Win3hs	Win3hs
**STAMP 2**		-0.909***		-1.258***		-2.346***
		(0.113)		(0.143)		(0.155)
**STAMP**	0.412***	1.144***	-0.265***	0.678***	1.461***	3.536***
	(0.0466)	(0.101)	(0.0545)	(0.119)	(0.0724)	(0.153)
**Selfciting**	0.242***	0.247***	0.263***	0.270***	0.251***	0.269***
	(0.0392)	(0.0393)	(0.0422)	(0.0424)	(0.0693)	(0.0700)
**International**	0.151***	0.147***	0.177***	0.173***	0.0893***	0.0730***
	(0.0155)	(0.0155)	(0.0171)	(0.0171)	(0.0243)	(0.0243)
**Author**	0.00594***	0.00619***	0.00501***	0.00525***	0.00937***	0.0106***
	(0.00166)	(0.00165)	(0.00186)	(0.00183)	(0.00264)	(0.00262)
	0.00516*	0.00432	0.00422	0.00322	0.00652	0.00446
	(0.00279)	(0.00280)	(0.00312)	(0.00313)	(0.00436)	(0.00438)
**Page**	0.00512***	0.00524***	0.00503***	0.00507***	-0.000350	2.27e-05
	(0.00138)	(0.00138)	(0.00151)	(0.00152)	(0.00236)	(0.00235)
**Reference**	0.00590***	0.00574******	0.00572***	0.00557***	0.00659***	0.00599***
	(0.000296)	(0.000298)	(0.000324)	(0.000327)	(0.000492)	(0.000498)
**Journal fixed effects**	YES	YES	YES	YES	YES	YES
**Year fixed effects**	YES	YES	YES	YES	YES	YES
**Constant**	-1.219***	-1.251***	-1.032***	-1.065***	-2.469***	-2.630***
	(0.107)	(0.107)	(0.113)	(0.113)	(0.196)	(0.198)
**Log likelihood**	-43239.193	-43206.051	-35986.022	-35946.158	-21292.616	-21173.799
***χ***^**2**^	5348***	5412***	4464***	4509***	2720***	2910***

*** p<0.01

** p<0.05

* p<0.1

Standard errors in parentheses

**Table 4:** Fixed effects negative binomial models: The percentage of hard sciences references and long-term citations (ten-year) (N = 3,240).

	(1)	(2)	(3)	(4)	(5)	(6)
	Win10all	Win10all	Win10hss	Win10hss	Win10hs	Win10hs
**STAMP2**		-2.158***		-1.155***		-1.429***
		(0.326)		(0.283)		(0.301)
**STAMP**	0.351***	2.109***	-0.446***	0.447*	0.455***	1.652***
	(0.125)	(0.287)	(0.105)	(0.239)	(0.122)	(0.276)
**Selfciting**	0.00262	0.00564	0.0343	0.0386	-0.0446	-0.0448
	(0.105)	(0.105)	(0.0781)	(0.0783)	(0.104)	(0.104)
**International**	0.316***	0.311***	0.199***	0.197***	0.161***	0.157***
	(0.0469)	(0.0468)	(0.0356)	(0.0355)	(0.0451)	(0.0451)
**Author**	0.0105***	0.0112***	0.00481*	0.00512*	0.0101***	0.0105***
	(0.00320)	(0.00324)	(0.00287)	(0.00289)	(0.00337)	(0.00341)
**Keyword**	0.0319***	0.0303***	0.0135	0.0131	0.0190*	0.0178*
	(0.0102)	(0.0102)	(0.00876)	(0.00875)	(0.0104)	(0.0104)
**Page**	-0.00584	-0.00560	0.00514*	0.00495*	0.00132	0.00163
	(0.00364)	(0.00363)	(0.00280)	(0.00280)	(0.00357)	(0.00356)
**Reference**	0.00713***	0.00656***	0.00544***	0.00521***	0.00535***	0.00493***
	(0.000871)	(0.000876)	(0.000714)	(0.000718)	(0.000844)	(0.000848)
**Journal fixed effects**	YES	YES	YES	YES	YES	YES
**Year fixed effects**	YES	YES	YES	YES	YES	YES
**Constant**	-1.883***	-1.946***	-0.742***	-0.765***	-1.247***	-1.298***
	(0.153)	(0.153)	(0.119)	(0.119)	(0.150)	(0.150)
**Log likelihood**	-8237.7057	-8220.1465	-8443.3449	-8434.827	-5811.9837	-5800.502
***χ***^**2**^	294.1***	340.6***	369.4***	377.6***	228.3***	251.7***

*** p<0.01

** p<0.05

* p<0.1

Standard errors in parentheses

The ratio of HS references has inverted U-shaped effect on citation counts in both Social Sciences articles and Humanities articles in a short-term citation window. To explore differences between Humanities and Social Science, we conduct the same regression procedure and provide estimated citations counts by dividing HSS articles into two subsamples according to their disciplinary properties, as reported in S1 Table in Supporting Information. Because there are only 187 observations of Humanities articles in 10-year citation window, we only focus on short-term citation counts. Obviously, inverted U-shaped relationship between the ratio of HS references and citation counts from HS and HSS is also found in both social sciences articles sample and the humanities one in a short-term citation window. For example, in social sciences articles, before reaching 63%, 27%, 75%, the percentage of HS references may increase overall citations, citations from HSS and those from HS.

### Robustness check

In this study, three strategies were utilized to test the robustness of our findings. The first one is to exclude the influence of articles which are more likely to be HS articles rather than HSS articles. As mentioned in Data and measurement, we try to assign a unique category to each sample article based on the category of journals where the article is published. However, we cannot deny that articles with research topics more similar to HS rather than HSS can also be published in HSS journals. This kind of articles may cite a number of references in HS to address HS research problems, and consequently receive more citations from HS. Therefore, these articles are actually HS articles to some extent. In this case, these articles may not be the ones belonging to HSS category we are interested in so that we eliminate them from our sample articles for a more robust estimation. Specifically, we excluded articles (accounting for 14.10% of the sample articles in a short-term citation window, and 10.24% of those in a long-term citation window) where there are more HS references than HSS references and which receive more citation counts from HS than HSS. Secondly, we regard the number of HS references (*STAMN*) as the explanatory variable rather than the ratio of HS references. Thirdly, given that the Poisson models are commonly used in the literature, we also fitted the xtpoisson models for a robustness check, as shown in S2 and S3 Tables in Supporting Information. Apparently, significant inverted U-shaped relationship consistently occurs in both a short term and a long term.

## Discussion and conclusions

The hypothesis in this study was supported by the empirical analysis. An inverted U-shaped relationship was observed between the ratio of HS knowledge cited and the number of citations HSS articles received. Firstly, the results show that absorbing more HS knowledge does not necessarily bring more citations to HSS papers. However, at the initial stage, citing more HS knowledge does bring more citations to HSS articles. One possible reason is that articles combining knowledge in both HSS and HS face more readers than those in a mono-discipline [18]. Another possible reason is that the prevalence of interdisciplinary research spurred more and more researchers to introduce interdisciplinary knowledge to their particular discipline. However, on condition that the ratio of HS knowledge is beyond a certain value, the very increase of HS knowledge in HSS articles undermines their impact. Furthermore, we observed that the turning point in the model using citations from HSS researchers as the dependent variable, is far smaller than the one using citations from HS researchers. It implies that integrating knowledge from HS into HSS research mainly attracts citations from HS researchers rather than HSS researchers. It may be interpreted that an excess of HS knowledge in HSS articles jeopardizes their readability. The turning point in the short-term citation window is smaller than that in the long-term citation window, pointing to the fact that the optimal proportion of HS references for high citations appears earlier in the short-term citation window.

It is identified that HSS research absorbed more and more HS knowledge. Government policy encouraged HSS researchers to integrate HS into their research fields. China has entered a critical period of reforming and opening up, being stuck in emerging tricky problems in the fields of political science, sociology and economics that remained to be solved by HSS. In many speeches of China’s leaders, boosting the interaction between HSS and HS was mentioned. In 2011, *Decision of the Central Committee of Communist Party of China on Some Major Issues Concerning Deepening the Reform of Culture to Promote the Development and Prosperity of Socialistic Culture* was adopted, encouraging HSS scientists not only to conduct interdisciplinary research within HSS but also to expand their horizons to HS. In addition, it is proved that searching more distant domains of knowledge can assist researchers in avoiding being trapped in inefficient local optima [60].

HS knowledge was unevenly distributed in HSS fields. In Social Sciences we found a stronger interest in HS knowledge than in Humanities. The rapid growth rate of Technology knowledge since 2008 in HSS implies its growing importance in recent years because with ever more accessible data and advanced information technology, HSS researchers became increasingly dependent on Technology.

Chinese HSS scholars are now inspired to blaze an interdisciplinary path to avoid the “ceiling phenomenon” in their research fields, widening their horizons to HS, hoping to conduct high-quality studies and gain more international citations. However, our analysis results proved that “more HS knowledge, more citations” is not always true. For researchers, it is not applicable to expand their scientific impact by simply increasing HS knowledge in their references, which may undermine citations instead. Moreover, facilitating the integration of HSS and HS also relies on smooth communications between scholars in HSS and HS fields.

For policy making, it is appropriate to emphasize how to integrate HS knowledge with HSS research fields rather than just encourage HSS scholars to learn from HS, given that citing more HS may be influenced by policy guidelines but not every research field in HSS is suitable for HS, which depends on specific research questions and targets. Furthermore, learning from HS should not be limited to copying a scientific theory without deliberation, or studying a mathematical/technological tool, but be expanded to generating new perspectives to understand and solve problems better based on the fundamental knowledge of HSS itself. Therefore, the research policy should be adjusted, guiding HSS scientists to study from HS with problem-oriented approaches.

From the perspective of funding agencies, HSS projects involving HS should be treated cautiously. Extracting knowledge from HS properly can improve scientific impacts of HSS and even knowledge flow; however, negative effects appear if HS knowledge is over-integrated into HSS research. Therefore, having preferences or prejudice for this kind of HSS research does not make sense. More attention should be paid to whether this kind of HSS research helps to understand or settle meaningful questions.

Although we used a collection of articles published by China, there is no evidence that China’s HSS exclusively features the inverse U-shaped relationship between the proportion of HS references in HSS articles and the number of citations they received. Nevertheless, whether the result is applicable to other counties or territories requires further studies.

As a bibliometric analysis, limitations are unavoidable in this study. First, we used only HS references as an indicator to measure knowledge flow from HS to HSS, which should be complemented by the investigation of other learning forms, such as collaboration or communication with HS scientists. Second, in addition to articles indexed in WoS, there are other important knowledge sources, such as books, which are not covered in this research.

## Supporting information

S1 Table Fixed effects negative binomial models: The percentage of hard sciences references and short-term citation (three-year) (N = 31,335). (PDF)

S2 Table Fixed effects poisson models in robustness check: The number of hard sciences references and short-term citation(three-year). (PDF)

S3 Table Fixed effects poisson models in robustness check: The number of hard sciences references and long-term citation(ten-year). (PDF)

S1 Data The data in 3-year citation window. (XLSX)

S2 Data The data in 5-year citation window. (XLSX)

S3 Data The original record of sampling articles. (XLSX)
