# Supplementary material for: Double-edged sword of interdisciplinary knowledge flow from hard sciences to humanities and social sciences: Evidence from China
Source: PLoS One. 2017 Sep 21;12(9):e0184977. doi: 10.1371/journal.pone.0184977 (PMC5608305; doi:10.1371/journal.pone.0184977)
Supplement: S1 Table — (PDF) [file pone.0184977.s001.pdf]

S1 Table. Fixed Effects Negative Binomial Models: the Percentage of Hard Sciences References and Short-Term Citation (three-year) (N=31,335).

	(1)	(2)	(3)	(4)	(5)	(6)
	Social Sciences articles(N=28,951)			Humanities articles(N=1374)		
	win3all	win3hss	win3hs	win3all	win3hss	win3hs
**STAMP2**	-0.894***[a]	-1.250***	-2.343***	-3.018***	-3.622***	-4.031***
	(0.113)[b]	(0.144)	(0.155)	(0.641)	(0.796)	(1.096)
**STAMP**	1.134***	0.670***	3.536***	2.800***	2.699***	5.052***
	(0.101)	(0.119)	(0.154)	(0.518)	(0.583)	(0.997)
**Selfciting**	0.251***	0.274***	0.268***	0.0516	-0.331	1.010
	(0.0394)	(0.0425)	(0.0702)	(0.408)	(0.483)	(0.704)
**International**	0.144***	0.172***	0.0677***	0.381***	0.342***	0.606***
	(0.0155)	(0.0172)	(0.0243)	(0.100)	(0.108)	(0.201)
**Author**	0.00801***	0.00626***	0.0131***	-0.00535	-0.00576	-0.00635
	(0.00159)	(0.00183)	(0.00245)	(0.00749)	(0.0115)	(0.0117)
**Keyword**	0.00378	0.00276	0.00392	0.0209	0.0291*	0.00467
	(0.00280)	(0.00313)	(0.00438)	(0.0159)	(0.0168)	(0.0369)
**Page**	0.00509***	0.00481***	5.82e-05	-0.00853**	-0.00755**	-0.0129
	(0.00138)	(0.00152)	(0.00236)	(0.00360)	(0.00375)	(0.00799)
**Reference**	0.00576***	0.00560***	0.00599***	0.00848***	0.00791***	0.0121***
	(0.000298)	(0.000327)	(0.000499)	(0.00198)	(0.00213)	(0.00413)
**Constant**	-1.248***	-1.063***	-2.628***	-0.981	-0.852	-13.21
	(0.107)	(0.113)	(0.198)	(0.693)	(0.690)	(517.0)
**Journal fixed effects**	YES	YES	YES	YES	YES	YES
**Year fixed effects**	YES	YES	YES	YES	YES	YES
**Log likelihood**	-43075.75	-35851.56	-21099.078	-1293.9145	-1144.5659	-354.26062
χ^2	5415***	4507***	2926***	218.1***	196.1***	94.28***

a. *** p<0.01, ** p<0.05, * p<0.1

b. Standard errors in parentheses
