# Supplementary material for: Double-edged sword of interdisciplinary knowledge flow from hard sciences to humanities and social sciences: Evidence from China
Source: PLoS One. 2017 Sep 21;12(9):e0184977. doi: 10.1371/journal.pone.0184977 (PMC5608305; doi:10.1371/journal.pone.0184977)
Supplement: S2 Table — (PDF) [file pone.0184977.s002.pdf]

S2 Table. Fixed Effects Poisson Models in Robustness Check: the Number of Hard Sciences References and Short-Term Citation(three-year).

	**(1)**	**(2)**	**(3)**	**(4)**	**(5)**	**(6)**
	Win3all	Win3all	Win3hss	Win3hss	Win3hs	Win3hs
**STAMN2**		-0.000433***		-0.000134***		-0.00121***
		(4.03e-05)		(3.94e-05)		(9.92e-05)
**STAMN**	0.00967***[a]	0.0290***	0.00346***	0.00899***	0.0211***	0.0734***
	(0.000865)[b]	(0.00169)	(0.00118)	(0.00192)	(0.00155)	(0.00358)
**Selfciting**	0.0806***	0.162***	0.118***	0.139***	0.0102	0.274***
	(0.0224)	(0.0234)	(0.0269)	(0.0275)	(0.0444)	(0.0482)
**International**	0.158***	0.159***	0.180***	0.180***	0.0251	0.0293
	(0.0101)	(0.0101)	(0.0118)	(0.0118)	(0.0198)	(0.0198)
**Author**	0.00866***	0.00842***	0.00680***	0.00674***	0.0130***	0.0123***
	(0.000951)	(0.000959)	(0.00110)	(0.00111)	(0.00227)	(0.00233)
**Keyword**	-0.00854***	-0.00953***	-0.0116***	-0.0118***	-0.00418	-0.00762*
	(0.00199)	(0.00199)	(0.00235)	(0.00235)	(0.00411)	(0.00415)
**Page**	0.00736***	0.00711***	0.00831***	0.00823***	0.0151***	0.0127***
	(0.000935)	(0.000933)	(0.00111)	(0.00111)	(0.00200)	(0.00201)
**Reference**	0.00462***	0.00464***	0.00518***	0.00517***	0.000742	0.000853*
	(0.000212)	(0.000211)	(0.000239)	(0.000239)	(0.000506)	(0.000504)
**Journal fixed effects**	YES	YES	YES	YES	YES	YES
**Year fixed effects**	YES	YES	YES	YES	YES	YES
**Observations**	26,242	26,242	25,226	25,226	22,454	22,454
**Log likelihood**	-54440.19	-54326.549	-40299.23	-40291.841	-20170.015	-19992.669
χ^2	13116***	13301***	9540***	9549***	3586***	3906***

a. *** p<0.01, ** p<0.05, * p<0.1

b. Standard errors in parentheses
