# Supplementary material for: Double-edged sword of interdisciplinary knowledge flow from hard sciences to humanities and social sciences: Evidence from China
Source: PLoS One. 2017 Sep 21;12(9):e0184977. doi: 10.1371/journal.pone.0184977 (PMC5608305; doi:10.1371/journal.pone.0184977)
Supplement: S3 Table — (PDF) [file pone.0184977.s003.pdf]

S3 Table. Fixed Effects Poisson Models in Robustness Check: the Number of Hard Sciences References and Long-Term Citation(ten-year).

	(1)	(2)	(3)	(4)	(5)	(6)
	Win10all	Win10all	Win10hss	Win10hss	Win10hs	Win10hs
**STAMN2**		-0.00146***		-0.000756***		-0.00272***
		(0.000123)		(0.000153)		(0.000196)
**STAMN**	0.00621***[a]	0.0396***	-0.00812***	0.00811**	0.0253***	0.0971***
	(0.00173)[b]	(0.00324)	(0.00220)	(0.00389)	(0.00287)	(0.00553)
**Selfciting**	-0.145***	-0.0893***	-0.0651**	-0.0428	-0.218***	-0.0371
	(0.0263)	(0.0268)	(0.0293)	(0.0297)	(0.0485)	(0.0493)
**International**	0.262***	0.258***	0.224***	0.223***	0.194***	0.199***
	(0.0119)	(0.0119)	(0.0131)	(0.0131)	(0.0225)	(0.0223)
**Author**	0.00545***	0.00569***	0.00390***	0.00400***	0.00836***	0.00911***
	(0.000783)	(0.000789)	(0.000861)	(0.000864)	(0.00187)	(0.00188)
**Keyword**	-0.00934**	-0.00928**	-0.0107***	-0.0107***	-0.0196***	-0.00791
	(0.00366)	(0.00366)	(0.00409)	(0.00409)	(0.00712)	(0.00673)
**Page**	0.00635***	0.00515***	0.00690***	0.00645***	0.0108***	0.00516***
	(0.00105)	(0.00105)	(0.00114)	(0.00115)	(0.00199)	(0.00193)
**Reference**	0.00814***	0.00805***	0.00802***	0.00799***	0.00561***	0.00512***
	(0.000291)	(0.000291)	(0.000315)	(0.000315)	(0.000604)	(0.000591)
**Journal Fixed Effects**	YES	YES	YES	YES	YES	YES
**Year Fixed Effects**	YES	YES	YES	YES	YES	YES
**Observations**	3,556	3,556	3,202	3,202	3,034	3,556
**Log likelihood**	-28382.645	-28307.257	-17047.146	-17034.179	-9046.4063	-8956.2099
χ^2	3902***	4062***	2630***	2658***	1263***	1528***

a. *** p<0.01, ** p<0.05, * p<0.1

b. Standard errors in parentheses
